# Synthesis, Structural and Magnetic Properties of BiFeO_3_ Substituted with Ag

**DOI:** 10.3390/ma18071453

**Published:** 2025-03-25

**Authors:** Maria Čebela, Pavla Šenjug, Dejan Zagorac, Igor Popov, Jelena Zagorac, Milena Rosić, Damir Pajić

**Affiliations:** 1 “Vinča” Institute of Nuclear Sciences, National Institute of the Republic of Serbia, University of Belgrade, Mike Petrovića Alasa 12-14, 11351 Belgrade, Serbia; dzagorac@vin.bg.ac.rs (D.Z.); jelena@vin.bg.ac.rs (J.Z.); 2 Faculty of Science, Department of Physics, University of Zagreb, Bijenička Cesta 32, 10000 Zagreb, Croatia; psenjug@phy.hr; 3 Center of Excellence “CextremeLab”, “Vinča” Institute of Nuclear Sciences, National Institute of the Republic of Serbia, University of Belgrade, Mike Petrovića Alasa 12-14, 11351 Belgrade, Serbia; 4 Institute for Multidisciplinary Research, University of Belgrade, Kneza Višeslava 1, 11030 Belgrade, Serbia; popov@ipb.ac.rs; 5 Institute of Physics, University of Belgrade, Pregrevica 118, 11080 Belgrade, Serbia

**Keywords:** bismuth ferrite, multiferroics, XRD, magnetoelectric effect, crystal structure prediction, bond valence calculations, density functional theory

## Abstract

Here, we report the hydrothermal synthesis of BFO (bismuth ferrite) and Bi_1−x_Ag_x_FeO_3_ (x = 0.01, 0.02) ultrafine nanopowders. The diffraction patterns show that all obtained particles belong to the *R3c* space group. On top of that, crystal structure prediction has been accomplished using bond valence calculations (BVCs). Several promising perovskite structures have been proposed together with experimentally observed modifications of BFO as a function of silver doping. Magnetization measurements were performed on BFO, both pure and substituted with 1% and 2% of Ag. The addition of Ag in BFO did not affect the Neel temperature, T_N_ = 630 K for all samples; instead, the influence of Ag was observed in the increase in the value and irreversibility of magnetization, which are usual characteristics of weak ferromagnetism. Our calculations based on density functional theory (DFT) are in agreement with the experimental finding of enhanced magnetization upon Ag doping of antiferromagnetic BFO, which is assigned to the perturbation of magnetic-type interactions between Fe atoms by Ag substitutional doping. Additionally, electronic and magnetic properties were studied for all phases predicted by the BVCs study. DFT predicted half-metallicity in the γ phase of BFO, which may be of great interest for further study and potential applications.

## 1. Introduction

After many years of practical and scientific interests, perovskite-type bismuth ferrites still attract enormous attention. This is due to their ferroelectric and antiferromagnetic arrangement in only one phase [1,2,3]. Due to the variety of their unique behaviors, they have attracted a wide range of applications such as optoelectronic and spintronic devices, data storage, chemical resistant sensors and photocatalytic activities [4,5,6,7,8,9]. Many examinations have been conducted on the substitution of bismuth ions in BiFeO_3_ by alkaline earth, transition and rare earth metals like Sr^2+^, Mn^3+^, Nb^3+^ and La^3+^ to name just a few of the numerous examples [10,11,12]. Perovskites with mixed metal oxides provide an important class of electric, ferroelectric, catalytic and magnetic materials [13]. From the magnetic and ferroelectric viewpoint, the nanosized ferrite particles with appropriate shapes usually display qualitatively new properties concerning their bulk counterparts, such as changes in magnetic transition temperatures, the emergence of superparamagnetism, size dependence of the saturation magnetization and coercivity, etc. [14,15].

Under standard conditions, the known modification of bismuth ferrite crystallizes in the perovskite structure type (α) and possesses a rhombohedral space group *R3c* (no. 161) [16,17,18,19].

The location of residual porosity, microstructure, particle size, grain growth behavior and grain boundary characteristics in sintered specimens play a crucial role in determining the electrical and magnetic properties of BiFeO_3_ [20,21,22,23]. Notably, introducing dopants in BiFeO_3_ materials can significantly impact their structure and properties. Recent studies have successfully fabricated various morphologies of Ag-substituted BiFeO_3_, including thin films [24,25,26,27], nanofilms [28], nanoparticles [29,30], nanopowders [31,32], nanocrystalline BFO [33] and bulk/polycrystalline ceramics [34,35,36,37].

The development of the secondary phase, weak electromagnetic coupling and weak magnetization are only some of the reasons for the limited technological application of bismuth ferrites. Doping is used to increase its multiferroic properties. Perovskite-type bismuth ferrites have the general formula of ABO_3_, which is complicated by doping with various elements in appropriate stoichiometric ratios. Doping leads to a charge imbalance in the system, which can be adjusted by creating oxygen vacancies, charge disproportionality of Fe^3+^ and Fe^5+^ or partial exchange of Fe^3+^ into Fe^4+^ [13]. The element we use as a dopant in this perovskite structure will occupy the A or B site or both of them (amphoteric) depending on its ionic radius, where if it is larger, it will occupy the A site, and if it is smaller, it will occupy the B site [38]. Silver ions are used as a dopant, and since they are much larger than Fe cations, Ag will occupy the A sites. If it were not so, partial substitution of Fe for Ag would affect the change in Fe valence. The perovskite structures are governed by the Goldschmidt tolerance factor *f* = (*r_A_* + *r_O_*)/√2(*r_B_* + *r_O_*) [39]. The stress in the structure is due to the mismatch between the equilibrium lengths of the A-O and B-O bonds. When the Goldschmidt tolerance factor is *f* = 1, it represents an unstressed cubic lattice. If *f* > 1 or *f* < 1, relaxed deformation occurs by distorting the structure from its ideal cubic shape. The Goldschmidt tolerance factor is 0.9309, which leads us to conclude that the observed nanometric structure could be stable.

In this study, we investigated the effects of Ag doping on the structural, electrical and magnetic properties of BiFeO_3_. To advance the understanding of BiFeO_3_’s magnetic behavior, we complemented experimental magnetization measurements with ab initio theoretical calculations. A central focus of this study was the intricate spin cycloid, a fundamental feature governing the multiferroic properties of BiFeO_3_, and the development of strategies for its precise control. The investigation further extended to a detailed analysis of the structure–property relationship under Ag doping, providing new insights into the role of compositional tuning. Additionally, predictive modeling enabled the identification of novel perovskite structures alongside a comprehensive evaluation of their electronic and magnetic properties. By integrating high-resolution synthesis, XRD characterization, magnetic analysis and theoretical predictions, this work offers a profound understanding of the impact of Ag substitution on the structural and magnetic evolution of BiFeO_3_, establishing a robust framework for tailoring its functional properties.

## 2. Materials and Methods

### 2.1. Synthesis and Structural Characterization of Bi_1−x_Ag_x_FeO_3_ (x = 0.01, 0.02) Nanopowders

Nanocrystalline powders of Bi_1−x_Ag_x_FeO_3_ were synthesized using the hydrothermal method, a well-established technique recognized for its energy efficiency, cost-effectiveness and ability to yield high-purity products with controlled morphology. High-purity precursors, including bismuth nitrate (Bi(NO_3_)_3_·5H_2_O), silver nitrate (AgNO_3_), iron nitrate (Fe(NO_3_)_3_·9H_2_O) and potassium hydroxide (KOH), were employed to ensure the reproducibility and phase purity of the final material. The synthesis involved dissolving stoichiometric amounts of Bi(NO_3_)_3_·5H_2_O, AgNO_3_ and Fe(NO_3_)_3_·9H_2_O in 40 mL of an 8 M KOH solution, followed by vigorous stirring for 30 min to promote homogeneous mixing and precursor reactivity. The resulting solution was then transferred into a Teflon-lined stainless steel autoclave and subjected to hydrothermal treatment at 200 °C under autogenous pressure for 6 h, facilitating crystallization under controlled conditions. Following the reaction, the system was cooled naturally to ambient temperature, and the precipitated powders were carefully collected. To eliminate residual reactants and potential secondary phases, the powders underwent multiple washing cycles involving centrifugation with distilled water. Subsequently, they were dispersed in ethanol and subjected to ultrasonication for 60 min to break up agglomerates and ensure uniform particle distribution. Finally, the purified powders were dried via controlled evaporation of ethanol using a heated mortar maintained at 60 °C, yielding nanocrystalline Bi_1−x_Ag_x_FeO_3_ with well-defined structural characteristics [40].

Multiple syntheses of bismuth ferrite doped with two distinct concentrations of Ag were successfully performed, yielding phase-pure samples in each case. This consistency underscores the high precision and reproducibility of the synthesis process. For magnetic characterization, a representative sample was carefully selected for each doping level to ensure that the measured properties accurately reflect the intrinsic magnetic behavior of the doped bismuth ferrite system.

The structure of the obtained powder was determined by X-ray powder diffraction on a Rigaku ULTIMA IV XRPD diffractometer (Tokyo, Japan) with Cu Kα1,2 radiations, at room temperature. Data for structural refinement were taken in the 2*θ* range 10–110°, with a step width of 0.02° 2*θ* and 5 s per step. The refinement was performed with the FullProf (version 5.90) [41] computer program which adopts the Rietveld calculation method. In the present approach, the grain size broadening was represented by a Lorentzian function, and strain broadening by a Gaussian function.

### 2.2. Magnetic Measurements

Magnetization was measured on the MPMS 5 SQUID magnetometer (Quantum Design Inc., San Diego, CA, USA) in a wide temperature range, from 2 K to 720 K, and in fields up to 5T. The high temperatures were realized using an oven option, which enabled the measurements at the high temperatures. To measure the temperature dependence of magnetization or the so-called zero-field-cooled (ZFC) and field-cooled (FC) magnetization curves of the compounds with the high magnetic phase transition temperature, such as pure BFO (T N ~630 K), correctly through the entire temperature range, it was necessary to use the following procedure. The samples were first cooled in zero field till 2 K and measured with the standard setup (pellet in the plastic straw) until T = 400 K; then, the samples were taken out and put on a quartz tube holder of the oven option and inserted back for the measurements on the high temperatures. After completing the ZFC curve, the FC magnetization was measured while cooling from 720 K to room temperature, where again the sample was removed and put in a standard setup for the continuation of the FC curve. For all the compounds, M(T) was measured in the constant field of 1 kOe. In addition to the M(T) measurements, the field dependence was measured at temperatures of 4 K and 300 K on a standard setup.

### 2.3. Crystal Structure Prediction (CSP) and Bond Valence Calculations (BVC)

Our general approach and methods of crystal structure prediction (CSP) and identification of structure candidates have been given in detail elsewhere [42,43,44,45], especially including the multidisciplinary approach and previous successful applications on perovskites [46,47,48,49]. The Structure Prediction Diagnostic Software (SPuDS V2.21.05.11) [49] was used to generate potential perovskite-related structure candidates in a silver-doped BiFeO_3_ chemical system. The SPuDS program is based on bond valence calculations (BVCs) and is used to predict the crystal structures of perovskites, including those distorted by tilting of symmetric octahedra or caused by Jahn–Teller distortions [46,47,48,49]. The stability of the predicted perovskite-related structures is determined using the global instability index (GII). The GII is obtained by comparing the calculated bond valence sums and the ideal formal valences, and it directly corresponds to the amount of the Ag quantity in BiFeO_3_ and the different Glazer tilt systems. The symmetry of the novel predicted structures was analyzed with the algorithms SFND [50] and RGS [51], all implemented in the KPLOT version 3 program [52]. The predicted structures were visualized using the VESTA 4.6.0 software [53].

### 2.4. Density Functional Theory (DFT) Calculations

We utilized SIESTA 5.2 [54] implementation of DFT to determine the atomic structure, electronic and magnetic properties of investigated crystal phases. Valence electrons were modeled using norm-conserving Troullier–Martins pseudopotentials [55], incorporating partial core corrections. A double-zeta basis set was utilized, and calculations for potentials and charge density were performed on a real-space grid with a mesh cutoff energy of 350 Ry, ensuring total energy convergence within 0.1 meV per unit cell during self-consistency procedures. Generalized-gradient approximation with Coulomb interaction potential, GGA+U, [56,57,58,59] was employed. At the same time, we fixed the Hubbard U_eff_ = 3.8 eV for Fe 3d orbital and U_eff_ = 0.95 for Fe 4s orbital in all calculations, which was successfully applied in our previous calculations [40]. The crystals were modeled using conventional unit cells that contain 30 atoms (for *α*- and *R*-type structures) or 40 atoms (for *β*-, *γ*-, T1- and T2-type structures). The Brillouin zone was sampled by following k-point grids using the Monkhorst–Pack sampling: 8 × 8 × 4 (*α*-type structure), 6 × 6 × 6 (*β* -type structure), 8 × 8 × 8 (*γ*-type structure), 8 × 8 × 4 (*R*-type structure), 6 × 8 × 6 (T1- and T2-type structures). Atomic structures were relaxed using the conjugate gradient method, where the structures were considered optimized when the maximal force on atoms dropped below 0.04 eV/Angstrom. We examined a ferromagnetic (FM) configuration along with three antiferromagnetic configurations labelled AFM-a, AFM-c and AFM-g by setting up initial atomic spin moments on Fe atoms, which were allowed to relax in self-consistent cycles.

## 3. Results

### 3.1. X-Ray Powder Diffraction (XRPD) and Rietveld Refinement

The most common way to clarify the structural changes caused by Ag doping is using Rietveld refinement. This procedure requires a structural model that is an approximation of the actual structure. The starting structural model for the rhombohedral R3cH (no.161) space group using a hexagonal setting was built up with crystallographic data reported in [60].

**Figure 1:**
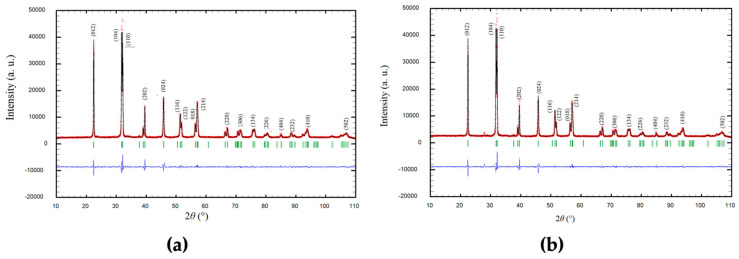
The structural refinement patterns of (**a**) Bi_0.99_Ag_0.01_FeO_3_ and (**b**) Bi_0.98_Ag_0.02_FeO_3_. A difference (observed–calculated) plot is shown beneath (blue line). Green stick marks above the different data indicate the reflection position. Red color denotes the experimental diffraction pattern.

The structure refinement was carried out on a FullProf program [61] which adopts the Rietveld calculation method. A pseudo-Voigt function was chosen as a profile function among profiles in the refinement program. The refinement of the background was performed using linear interpolation between 56 selected points. The best fit between the calculated and observed X-ray diffraction pattern is shown in Figure 1a,b, where all allowed Bragg reflections are shown by vertical bars. Inspecting the difference between the experimental and calculated profiles indicates good agreement. Besides the difference curve (blue line), the good refinement is proven by the R (reliability) factors that have values between 1.7 and 2.7.

**Figure 2:**
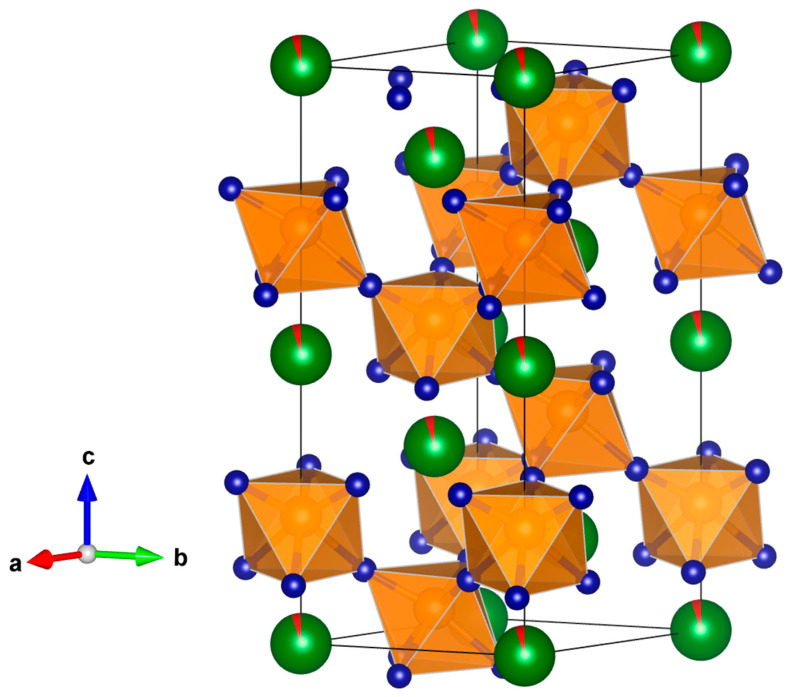
The refined silver-doped *α*-BiFeO_3_ structure in the rhombohedral R3cH (no.161) space group using a hexagonal setting. Green spheres correspond to Bi atoms, while red color corresponds to silver doping, blue atoms correspond to oxygen and orange atoms correspond to iron, represented by Fe-O octahedra.

The crystal structures of Bi_1−x_Ag_x_FeO_3_ (x = 0.01, 0.02) are shown in Figure 2.

The results of the Rietveld refinement, including unit cell parameters, atomic positions, and average bond distances, are given in Table 1. According to [62], the ionic radii of the Ag^+^ cation in coordination VI is 1.15 Å, and for the Bi^3+^ in the same coordination is 1.03 Å. Silver, instead of bismuth in the crystal lattice, will increase the unit cell parameters. In Table 1, we can follow the effect of entering silver in the structure of BiFeO_3_ by following values of the unit cell parameters, unit cell volume, and bond lengths. As expected, both investigated compounds have higher values for those parameters in comparison with undoped BiFeO_3_. Furthermore, we can see the difference between Bi_0.98_Ag_0.02_FeO_3_ where 2% of silver in the structure further increases the above-mentioned parameters in Bi_0.98_Ag_0.02_FeO_3_ if compared with Bi_0.99_Ag_0.01_FeO_3_. The changes in the crystal structure indicate the presence of silver in the structure, while the values of the occupation factors during Rietveld refinement were fixed to the nominal compositions of the powders.

### 3.2. CSP of Silver-Doped BiFeO_3_ Perovskites

A crystal structure prediction study was carried out in a bismuth ferrite system as a function of Ag concentration. Calculations were accomplished using bond valence calculations (BVCs) and SPuDS code to find possible additional structure candidates besides experimentally observed α-phase with perovskite structure type. Ten additional perovskite-related structure candidates were predicted, five with 0.1% silver in BFO and five with 0.2% Ag in BFO. Moreover, we predicted 0.5% Ag in BFO and generated an additional five structure candidates. These predicted structures are ranked according to the global instability index (GII) and tilt system and summarized in Table 2 (full structural data are presented in the Supporting Information).

**Figure 3:**
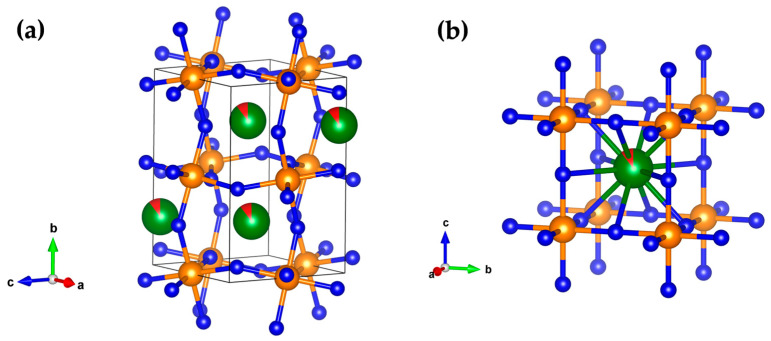
Visualization of the predicted: (**a**) *β*-type of structure in the space group Pnma (no. 62); (**b**) *γ*-type in the cubic Pm-3m (no. 221) space group, in the Bi_0.98_Ag_0.02_FeO_3_ compound.

The most stable BiFeO_3_ perovskite structure with silver doping, according to the GII criterion (Table 2), is the *β*-type in the space group Pnma. This is in agreement with previous experimental and theoretical data where the *β*-type structure was found in doped bismuth ferrite [30,31,36,40,62]. The GII ranging of the *β*-type structure increased from 0.02601 to 0.03542 at 2% Ag doping in BFO, indicating less stability with a higher level of silver dopant in BFO. The predicted *β* modification in the 2% Ag-doped BFO is shown in Figure 3a.

On the other hand, the *γ*-type of structure is the candidate with the highest level of the GII index, but the GII index is reduced with the increase in the Ag dopant, indicating that increasing silver concentration could stabilize this phase. This will be further confirmed later on in our DFT calculations (See Section 3.3). The gamma phase shows the highest cubic symmetry (Pm-3m (no. 221) space group) where the iron atoms are coordinated by six O atoms forming a perfect octahedron (Figure 3b). The *γ*-type modification was found in the experiments and theoretical studies in pure BiFeO_3_ at high temperatures, [6,63,64] and was also found in our previous research on pure BFO [40], indicating the possible existence of the gamma phase in the silver-doped bismuth ferrite.

**Figure 4:**
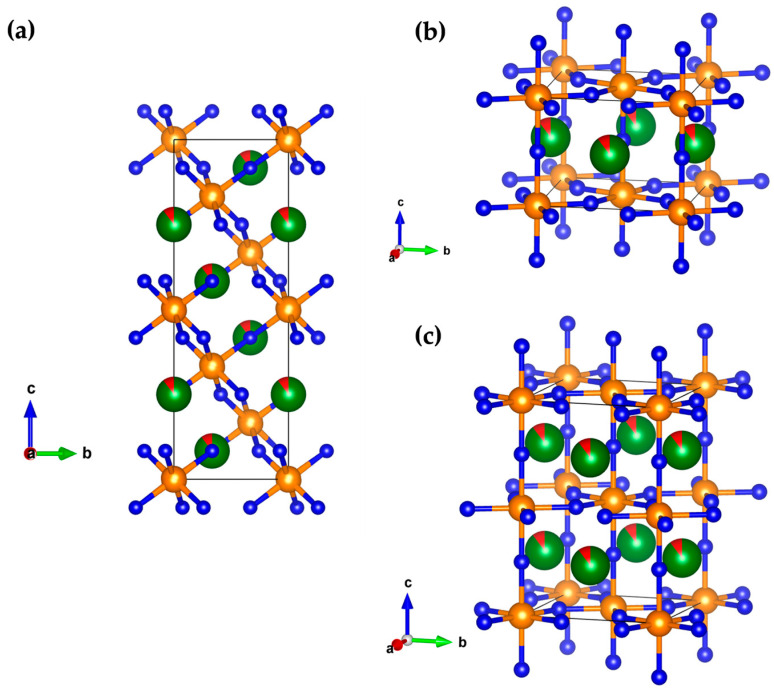
Visualization of the predicted: (**a**) *R*-type in the rhombohedral R-3c (no. 167) space group; (**b**) T1-type structure appearing in the space group P4/mbm (no. 127); (**c**) T2-type appearing in the I4/mcm (no. 140) space group in the Bi_0.98_Ag_0.02_FeO_3_ compound.

The next three predicted silver-doped structures in BFO, the *R*-, T1- and T2-type, are almost the same according to the GII index regardless of silver concentration, indicating the same possibility of synthesis between the beta and the gamma phase. The *R*-type appears in the rhombohedral R-3c (no. 167) space group (Figure 4a), structurally related to the experimentally observed alpha phase, making the *R*-type a bit more favorable in silver-doped bismuth ferrite. This concurs with previous experimental and theoretical research on pure BiFeO_3_ where high-temperature rhombohedral modification of BiFeO_3_ was identified [65,66]. On the other hand, both tetragonal T1 and T2 structures are closely related, where T2 modification appears as a polytype of T1 [67] (Figure 4b,c). Still, both phases are easily distinguishable by symmetry, where the T1 type appears in the P4/mbm (no. 127) space group, and the T2 type shows I4/mcm (no. 140) space group. Previous literature data on various tetragonal modifications in pure BiFeO_3_ at high temperatures [6,40,68,69] indicate that both T1- and T2-phases have a high potential for experimental synthesis in silver-doped BiFeO_3_. In order to explore higher doping levels of Ag in BFO, we predicted these perovskite structure candidates in the Bi_0.95_Ag_0.05_FeO_3_ compound. However, very small changes in structure and unit cell parameters were detected, implying that larger amounts of silver do not have a significant impact on the crystal structure of BFO (full structural data are presented in the Supporting Information).

### 3.3. Electronic and Magnetic Properties—Density Functional Theory Study

Using the structures predicted by the BVCs method, we analyzed the electronic and magnetic properties of the doped materials. To investigate the doped systems, we substituted one Bi atom with one Ag atom per conventional unit cell. This corresponds to doping of 12.5% (1/8) for *α*- and *R*-type structures and 16.6% (1/6) for remaining structural types. Unit cells that correspond to smaller, closer to experimental, doping levels are significantly larger than the unit cells which we used in the DFT calculations. Larger unit cells and the correspondingly larger number of atoms per unit cell significantly increase the number of degrees of freedom, which made geometry optimizations difficult and, for most phases, unachievable for the quantum method; atomic forces or even electron density could not converge below desired tolerance levels. However, differences between bond lengths of the doped materials predicted by the BVCs method for doping levels of 5% and 12.5% do not exceed 0.26%, which fairly justifies the utilization of the smaller unit cells. Additionally, the BVCs study indicated the stabilization of a studied crystal, particularly the γ phase, by an increase in Ag doping. Our DFT calculations, as will be shown below, confirmed this indication for a significantly larger doping concentration than that considered in BVCs. We proceeded with DFT calculations in the following steps. First, using the conjugate gradient method, we optimized the atomic structure of the undoped crystals. The optimization was performed with atomic spins initially set up in a ferromagnetic FM, or one of the three antiferromagnetic spin orders, AFM-a, AFM-c or AFM-g. The spin orders were determined by initial spins on Fe atoms. Second, we substituted one Bi atom with one Ag atom in the optimized undoped structure. After setting up the atomic spins to the previously obtained values in undoped structures, the conjugate gradient optimization was conducted for the doped structures. Table 3 shows the energy released upon the substitution of one Bi atom by one Ag atom in the unit cell. It is calculated from the expression E_dop_ = E(doped) + E(Bi) − E(undoped) − E(Ag), where E(doped) is the energy of the doped crystal, E(undoped) is the energy of the pristine crystal and E(Bi) and E(Ag) are energies of isolated Bi and Ag atoms. For the crystals with theoretically considered high levels of Ag doping, our DFT calculations indicate that only three phases can be substitutionally doped: *γ*, T1 and T2 with ground states having FM, AFM-c and AFM-a spin textures, respectively. Note that, e.g., molecular dynamics simulations (MDSs) would be essential to study the thermodynamic stability of these phases at finite temperatures, from, for example, the Lindemann index. However, MDSs are very “expensive” for investigated systems, in which atomic forces and density matrices were difficult to converge in self-consistent field iterations even in the conjugate gradient structural optimizations (essentially presenting quasi-dynamics of atomic structures at the absolute zero).

The spins were allowed to relax during self-consistent field iterations. For all investigated phases, the initial spin configurations were retained, while the obtained values of atomic spins are listed in Table S1 of the provided Supplementary Material. Two conclusions can be derived from Table S1:

1. Doping reduces the magnetization in the FM-ordered systems of all crystals by around 5% except for the R phase, for which magnetization is reduced by 7%.
2. In undoped systems, spins are perfectly compensated in AFM magnetic orders, whereas Ag doping induces net spins on Fe and O atoms in AFM magnetic textures.

**Figure 5:**
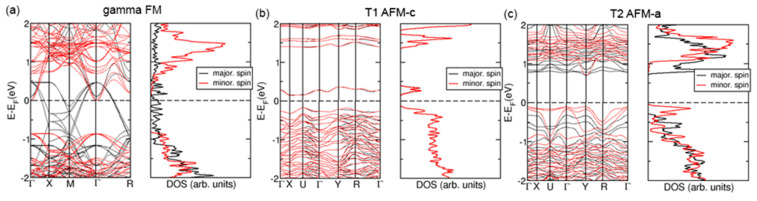
Electronic band structure (BS) and density of states (DOS) for ferromagnetic *γ* phase (**a**), T1 phase with AFM-c spin texture (**b**) and T2 phase with AFM-a spin texture (**c**). The figure on the left side of each panel represents BS, while the right-hand side figure of each panel shows DOS. The vertical scale of DOS figures matches the ones of BS. Features of majority spins are depicted by black lines, while those of minority spins are in red. Dash lines indicate Fermi’s energy.

Next, we show the electronic structure of the three systems with theoretically feasible high Ag doping, ferromagnetic *γ*-type structure, T1-type structure with AFM-c and T2-type structure with AFM-a spin textures, which are in ground electronic states according to our DFT calculations. Electronic band structures (BSs) and electronic density of states (DOS) for these phases are represented in Figure 5 in Panels (a), (b) and (c), respectively. The band structure of the ferromagnetic *γ*-type structure has mixed features of metal, semi-metal and half-metal. A band with a relatively large dispersion of about 2 eV starts at the center of the Brillouin zone (Γ-point at Γ-X segment) and crosses the Fermi level close to the M point. At the segment M-Γ-R, the bottom of the conduction band is placed below the top of the valence band, indicating the semi-metallic nature of the crystal. The majority spin characterizes the energy range from around −1 eV below the Fermi level to the Fermi level, indicating the half-metallic nature of the material in the *γ*-type structure. The T1 phase is nearly perfectly compensated antiferromagnet with AFM-c spin texture. It is also a narrow-gap semiconductor, with a direct gap of around 0.5 eV. In contrast, the T2-type structure with AFM-a spin texture is a semiconductor with a band gap of about 0.8 eV. Its valence band is characterized primarily by minority spin, whereas the conduction band has predominantly majority spin. The T2 phase has an indirect band gap, with the top of the valence band at the Γ-point and the bottom of the condition band at the Y point. We obtained electronic BS and DOS also for *α*, *β* and R phases and all combinations of spin textures. These are included in Figures S1 and S2 of the Supplementary Material.

### 3.4. Magnetic Properties

**Figure 6:**
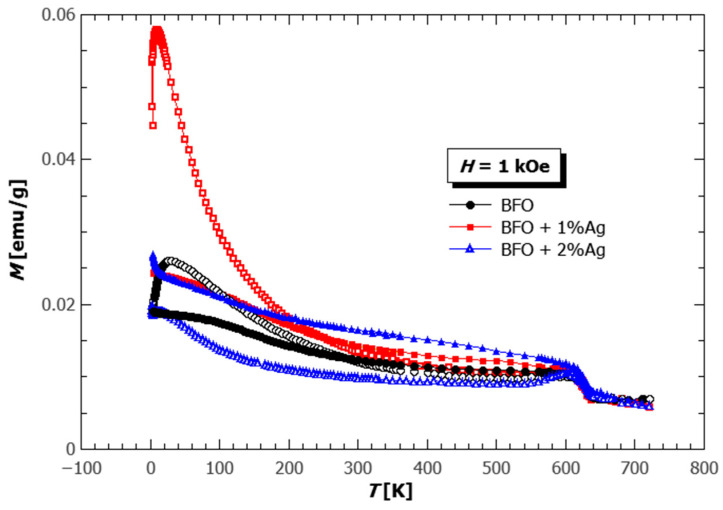
The temperature dependence of magnetization was measured in the magnetic field of 1 kOe on the pellets of pure BFO (black circles), BFO with 1% Ag (red rectangles) and 2% Ag (blue triangles). The empty symbols represent the ZFC, and the full symbols FC magnetization curves.

The temperature dependence of magnetization measured on the pellets of pure BFO, and BFO with 1% and 2% of Ag is shown in Figure 6. It can be seen that the incorporation of Ag increases the value and the irreversibility of magnetization but does not change the temperature of the magnetic phase transition (T_N_ = 630 K). Our DFT calculations are in agreement with the experimental finding of magnetization increase by Ag doping in AFM spin orders, with spins localized at Fe and O atoms. While pure BFO is known to be an antiferromagnet with the spin cycloid magnetic order [70], often, the weak ferromagnetic moment can be found due to the microstructure of the samples. Very rarely, it is synthesized in monocrystal form, which would show the peak in the magnetization at the transition temperature, and more often it is found to be in the form of nanoparticles, where the non-compensated surface spins and the breaking of spin spiral periodicity (period 62 nm) attribute to the appearance of the magnetic moment. The substitution of nonmagnetic Ag enhances the development of the weak ferromagnetic moment, and the irreversibility of the magnetization increases as it disturbs the antiferromagnetic interaction of the Fe ions. A similar effect is also found in the substitution with other elements [71,72,73].

A peculiar behavior is observed in pure BFO and BFO with 1% of Ag, where the crossing of ZFC and FC curves is present around 280 K and 250 K, respectively. Such behavior was already observed and can be attributed to different origins such as the competition of the interactions between Fe in different magnetic sublattices spin freezing in surface layers of small particles and other types of canting in the antiferromagnetic spin cycloids, etc. [74,75]. Only the former origin was attainable by our DFT calculations since we modeled the systems as infinite crystals (i.e., without surfaces), and we performed collinear spin calculations that do not take into account the spin canting in an antiferromagnet.

**Figure 7:**
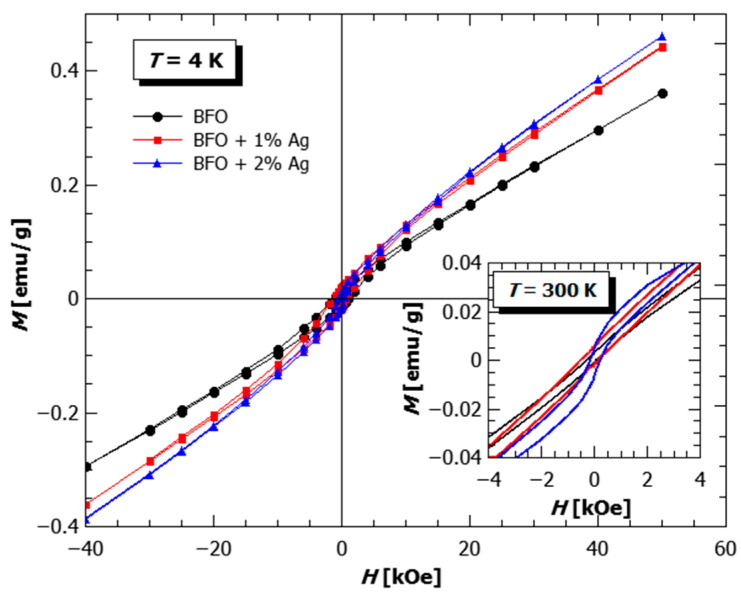
Field dependence of magnetization at 4 K and 300 K (inset), measured on the pellets of pure BFO (black circles), BFO with 1% Ag (red rectangles) and 2% Ag (blue triangles).

From the field dependence of magnetization at 4 K and 300 K (Figure 7), additional conclusions can be drawn in the same direction. The substitution of Ag increases the value of magnetization, which is expressed in the larger changes at the lowest fields and the additional appearance of the S-shape is superimposed on the antiferromagnetic linear dependence, which is characteristic of the weak ferromagnetic behavior. The pronounced weak ferromagnetic hysteresis is found in the BFO with 2% of Ag also at room temperature, while 0% Ag and 1% Ag curves resemble much more the shape of antiferromagnetic MH loops. This shows that even the addition of only 2% of a nonmagnetic substituent can produce a weak ferromagnetic response at ambient temperatures, thereby opening the possibilities of developing such materials towards eventual applications in different multiferroic devices.

## 4. Conclusions

A comprehensive multidisciplinary study was conducted to investigate the impact of doping BiFeO_3_ with Ag atoms. Nanocrystalline powders of BiFeO_3_ and Bi_1−x_Ag_x_FeO_3_ (with x = 0.01 and 0.02) were synthesized using the hydrothermal method. Samples doped with 1 and 2 atomic percent (at. %) of Ag were characterized using powder X-ray diffraction, and the structure refinement was carried out on a FullProf program, which adopts the Rietveld calculation method. The doped BiFeO_3_ samples, regardless of the Ag concentration, exhibited the α-phase structure in the space group *R3cH*. To explore additional possible structures in Ag-doped BiFeO_3_, bond valence calculations (BVCs) were employed for structure prediction. As a result, several structure candidates, including *α*-, *β*-, *γ*-, *R*-, T1 and T2 structures, were predicted in various Ag-doped BiFeO_3_ systems using BVCs. We studied the electronic and magnetic properties of these structures using DFT. Our DFT results are in agreement with the experimental finding of magnetization increase upon Ag doping in antiferromagnetically ordered structures and the possibility to dope the *γ* phase with a high Ag dopant concentration. Additionally, DFT calculations predict the semiconducting properties of antiferromagnetic T1 and T2 phases. More importantly, the half-metallic property of the *γ*-type structure that DFT calculations predicted, as well as the induced weak ferromagnetism with light doping, may be of great interest for further research due to its potential physical consequences and applications in future nanodevices.

## Figures and Tables

**Table 1:** Refined structure parameters for Bi_0.99_Ag_0.01_FeO_3_ and Bi_0.98_Ag_0.02_FeO_3_ in comparison with pure BiAgFeO_3_.

Sample	Structure Parameters	Distances
Bi_0.99_Ag_0.01_FeO_3_	*a* = 5.57708(6) *c* = 13.8667(3) *V* = 373.52 Å^3^ Bi/Ag 0 0 0 Fe 0 0 0.2170(2) O 0.570(1) 0.027(1) 0.9512(5)	3 × (Bi-O) = 2.5651(19) Å 3 × (Bi-O) = 2.227(6) Å Average = 2.3963 Å 3 × (Fe-O) = 2.002(4) Å 3 × (Fe-O) = 2.045(5) Å Average = 2.0231 Å
Bi_0.98_Ag_0.02_FeO_3_	*a* = 5.57765(6) *c* = 13.8682(3) *V* = 373.64 Å^3^ Bi/Ag 0 0 0 Fe 0 0 0.2208(2) O 0.562(2) 0.022(1) 0.9533(6)	3 × (Bi-O) = 2.586(3) Å 3 × (Bi-O) = 2.262(8) Å Average = 2.4244 Å 3 × (Fe-O) = 1.963(5) Å 3 × (Fe-O) = 2.075(7) Å Average = 2.0191 Å
BiFeO_3_ [1]	*a* = 5.5767(3) *c* = 13.8639(8) *V* = 373.40 Å^3^ Bi 0 0 0 Fe 0 0 0.2208(2) O 0.4478(4) 0.0196(5) 0.9522(2)	3 × (Bi-O) = 2.533(3) Å 3 × (Bi-O) = 2.265(3) Å Average = 2.3989 Å 3 × (Fe-O) = 1.957(5) Å 3 × (Fe-O) = 2.101(7) Å Average = 2.0289 Å

**Table 2:** Computed values of the GII and tilt system of the most promising predicted structure candidates in the (a) Bi_0.99_Ag_0.01_FeO_3_; (b) Bi_0.98_Ag_0.02_FeO_3_ system. Calculations were carried out using the BVCs method.

Composition	Bi_0.99_Ag_0.01_FeO_3_		
Modification	Space Group	Tilt System	GII
*β*-type	*Pnma*	a^−^b^+^a^−^	0.02601
*R*-type	*R*-3*c*	a^−^a^−^a^−^	0.13907
T1-type	*P*4/*mbm*	a^0^a^0^c^−^	0.14216
T2-type	*I*4/*mcm*	a^0^a^0^c^−^	0.14216
*γ*-type	*Pm*-3*m*	a^0^a^0^a^0^	0.76254
	Bi_0.98_Ag_0.02_FeO_3_		
*β*-type	*Pnma*	a^−^b^+^a^−^	0.03542
*R*-type	*R*-3*c*	a^−^a^−^a^−^	0.14136
T1-type	*P*4/*mbm*	a^0^a^0^c^−^	0.14433
T2-type	*I*4/*mcm*	a^0^a^0^c^−^	0.14433
*γ*-type	*Pm*-3*m*	a^0^a^0^a^0^	0.75986

**Table 3:** Energy (eV/unit cell) for substitution of one Bi atom by one Ag atom in the unit cell. The doping energy is calculated using the following formula: E_dop_ = E(doped) + E(Bi) − E(undoped) − E(Ag). The fields marked in blue indicate the doping energies in the ground states.

Phase	FM	AFM-a	AFM-c	AFM-g
***α***	0.103	2.351	N/A	N/A
***β***	1.357	1.639	1.737	2.077
***γ***	−0.350	0.127	0.704	1.531
**R**	0.102	1.623	N/A	N/A
**T1**	−0.383	−0.294	−0.609	−0.133
**T2**	−0.068	−0.353	1.456	−0.073

## Data Availability

The original contributions presented in this study are included in the article/Supplementary Material. Further inquiries can be directed to the corresponding authors.

## Supplementary Materials

The following supporting information can be downloaded at: https://www.mdpi.com/article/10.3390/ma18071453/s1, Figure S1: Electronic band structure and density of states for all combinations of alpha, beta, gamma and R phases on one hand and FM, AFM-a, AFM-c and AFM-g spin orders on the other hand; Figure S2: Electronic band structure and density of states for all combinations of T1 and T2 phases on one hand and FM, AFM-a, AFM-c and AFM-g spin orders on the other hand. Table S1: Calculated values of the global instability index (GII) and tilt system of the most promising Bi_0.99_Ag_0.01_FeO_3_ modifications using the bond valence calculations (BVCs) method.; Table S2: Structural data of the most promising Bi_0.99_Ag_0.01_FeO_3_ perovskite modifications were calculated using the BVCs method; Table S3: Calculated values of the global instability index (GII) and tilt system of the most promising Bi_0.98_Ag_0.02_FeO_3_ modifications using the bond valence calculations (BVCs) method; Table S4: Structural data of the most promising Bi_0.98_Ag_0.02_FeO_3_ perovskite modifications were calculated using the BVCs method; Table S5 Atomic spins (in units of Bohr magneton) were obtained by DFT calculations for each of the undoped or Ag-doped phases for FM, AFM-a, AFM-c and AFM-g spin textures. For undoped crystals, the Ag atom is not present and so spin on the Ag atom is not available (N/A). Data for Alpha and R phases in AFM-c and AFM-g spin configurations are not available as well, as these results could not converge.
