# Supplementary material for: Synthesis, Structural and Magnetic Properties of BiFeO3 Substituted with Ag
Source: Materials (Basel). 2025 Mar 25;18(7):1453. doi: 10.3390/ma18071453 (PMC11989932; doi:10.3390/ma18071453)
Supplement: Supplementary file 1 [file materials-18-01453-s001.zip › materials-3515754-supplementary.pdf]

Synthesis, structural and magnetic properties of $BiFeO_3$ substituted with Ag

Maria Čebela [1,2]*, Pavla Šenjug [2], Dejan Zagorac [1,3], Igor Popov [4,5], Jelena Zagorac [1,3], Milena Rosić [1]*, and Damir Pajić [2]*

[1] "Vinča" Institute of Nuclear Sciences, National Institute of the Republic of Serbia, University of Belgrade, Mike Petrovića Alasa 12-14, 11351 Belgrade, Serbia; mcebela@vin.bg.ac.rs; dzagorac@vin.bg.ac.rs; jelena@vin.bg.ac.rs; mrosic@vin.bg.ac.rs;
[2] University of Zagreb, Faculty of Science, Department of Physics, Bijenička cesta 32, 10000, Zagreb, Croatia; mcebela@vin.bg.ac.rs; dpajic@phy.hr; psenjug@phy.hr;
[3] Center of Excellence '"CextremeLab", "Vinča" Institute of Nuclear Sciences, National Institute of the Republic of Serbia, University of Belgrade, Mike Petrovića Alasa 12-14, 11351 Belgrade, Serbia; mcebela@vin.bg.ac.rs; dzagorac@vin.bg.ac.rs; jelena@vin.bg.ac.rs;
[4] Institute for Multidisciplinary Research, University of Belgrade, Kneza Višeslava 1, 11030, Belgrade, Serbia; popov@ipb.ac.rs;
[5] Institute of Physics, University of Belgrade, Pregrevica 118, 11080, Belgrade, Serbia; popov@ipb.ac.rs;
* Correspondence: mcebela@vin.bg.ac.rs (M.Č.); mrosic@vin.bg.ac.rs (M.R.); dpajic@phy.hr (D.P.);

Supporting Information

Table S1. Calculated values of the global instability index (GII) and tilt system of the most promising $Bi_{0.99}Ag_{0.01}FeO_3$ modifications using the bond valence (BVC) method.

$Bi_{0.99}Ag_{0.01}FeO_3$ Modification	Space group	Tilt system	GII
β-type	*Pnma*	$a^-b^+a^-$	0.02601
R-type	*R*-3*c*	$a^-a^-a^-$	0.13907
T_1-type	P4/*mbm*	$a^0a^0c^–$	0.14216
T_2-type	*I*4/*mcm*	$a^0a^0c^–$	0.14216
γ-type	*Pm*-3*m*	$a^0a^0a^0$	0.76254

Table S2. Structural data of the most promising $Bi_{0.99}Ag_{0.01}FeO_3$ perovskite modifications were calculated using the BVC method.

Modification, space group, Wyckoff position and occupancy	Cell parameters(Å), unit-cell volume (Å^3) and fractional coordinates
β-type	*a*=5.601, *b*=7.766, *c*=5.396
Pnma (no. 62)	*V*=234.69
Bi 4*c* (0.99)	0.5596 1/4 0.5205
Ag 4*c* (0.01)	0.5596 1/4 0.5205
Fe 4*b* (1.0)	1/2 0 0
O1 4*c* (1.0)	-0.0180 1/4 0.4016
O2 8*d* (1.0)	0.2957 0.0492 0.7008
R-type	*a*=5.426, *c*=13.964
R-3c (no. 167)	*V*=356.05
Bi 6*a* (0.99)	0 0 ¼
Ag 6*a* (0.01)	0 0 1/4

Fe1 6*b* (1.0)	2/3 1/3 1/3
O 18*e* (1.0)	0.4070 0 1/4
T1-type	*a*=5.337, *c*=4.031
P4/mbm (no. 127)	V=114.83
Bi 2*c* (0.99)	0 1/2 1/2
Ag 2*c* (0.01)	0 1/2 1/2
Fe 2*a* (1.0)	0 0 0
O1 2*b* (1.0)	0 0 1/2
O2 4*g* (1.0)	0.1562 0.6562 0
T2-type	*a*=5.337, *c*=8.062
I4/mcm (no. 140)	*V*=229.65
Bi 4*b* (0.99)	0 1/2 1/4
Ag 4*b* (0.01)	0 1/2 1/4
Fe 4*c* (1.0)	0 0 0
O1 4*a* (1.0)	0 0 1/4
O2 8*h* (1.0)	0.1562 0.6562 0
γ-type	*a*=4.031
Pm-3m (no. 221)	*V*=65.50
Bi 1*b* (0.99)	1/2 1/2 1/2
Ag 1*b* (0.01)	1/2 1/2 1/2
Fe 1*a* (1.0)	0 0 0
O 3*d* (1.0)	0 0 1/2

Table S3. Calculated values of the global instability index (GII) and tilt system of the most promising $Bi_{0.98}Ag_{0.02}FeO_3$ modifications using the bond valence (BVC) method.

$Bi_{0.98}Ag_{0.02}FeO_3$ Modification	Space group	Tilt system	GII
β-*type*	*Pnma*	$a^-b^+a^-$	0.03542
R-type	*R*-3*c*	$a^-a^-a^-$	0.14136
T1-type	*P*4/*mbm*	$a^0a^0c^-$	0.14433
T2-type	*I*4/*mcm*	$a^0a^0c^-$	0.14433
γ-type	*Pm*-3*m*	$a^0a^0a^0$	0.75986

Table S4. Structural data of the most promising $Bi_{0.98}Ag_{0.02}FeO_3$ perovskite modifications were calculated using the BVC method.

Modification, space group, Wyckoff position and occupancy	Cell parameters(Å), unit-cell volume (Å^3) and fractional coordinates
β-type	a=5.601, b=7.766, c=5.395
Pnma (no. 62)	V=234.664
Bi 4*c* (0.98)	0.5597 1/4 0.5206
Ag 4*c* (0.02)	0.5597 1/4 0.5206
Fe 4*b* (1.0)	1/2 0 0
O1 4*c* (1.0)	-0.0180 1/4 0.4015
O2 8*d* (1.0)	0.2957 0.0492 0.7008
R-type	a=5.427, c=13.964
R-3c (no. 167)	V=356.093
Bi 6*a* (0.98)	0 0 ¼
Ag 6*a* (0.02)	0 0 ¼
Fe1 6*b* (1.0)	2/3 1/3 1/3
O 18*e* (1.0)	0.4071 0 1/4
T_1-type	a=5.338, c=4.031
P4/mbm (no. 127)	V=114.839
Bi 2*c* (0.98)	0 1/2 ½
Ag 2*c* (0.02)	0 1/2 1/2
Fe 2*a* (1.0)	0 0 0
O1 2*b* (1.0)	0 0 1/2
O2 4*g* (1.0)	0.1562 0.6562 0
T_2-type	a=5.338, c=8.062
I4/mcm (no. 140)	V=229.683
Bi 4*b* (0.98)	0 1/2 1/4
Ag 4*b* (0.02)	0 1/2 1/4
Fe 4*c* (1.0)	0 0 0
O1 4*a* (1.0)	0 0 1/4
O2 8*h* (1.0)	0.3438 0.8438 0
γ-type	*a*=4.031
Pm-3m (no. 221)	*V*=65.50
Bi 1*b* (0.98)	1/2 1/2 1/2
Ag 1*b* (0.02)	1/2 1/2 1/2
Fe 1*a* (1.0)	0 0 0
O 3*d* (1.0)	0 0 1/2

Table S5. Calculated values of the global instability index (GII) and tilt system of the most promising $Bi_{0.95}Ag_{0.05}FeO_3$ modifications using the bond valence (BVC) method.

**$Bi_{0.95}Ag_{0.05}FeO_3$ Modification**	**Space group**	**Tilt system**	**GII**
β-type	*Pnma*	$a^-b^+a^-$	0.05654
R-type	*R*-3*c*	$a^-a^-a^-$	0.14799
T_1-type	*P*4/*mbm*	$a^0a^0c^-$	0.15061
T_2-type	*I*4/*mcm*	$a^0a^0c^-$	0.15061
γ-type	*Pm*-3*m*	$a^0a^0a^0$	0.75174

Table S6. Structural data of the most promising $Bi_{0.95}Ag_{0.05}FeO_3$ perovskite modifications were calculated using the BVC method.

**Modification, space group, Wyckoff position and occupancy**	**Cell parameters(Å), unit-cell volume (Å³) and fractional coordinates**
β-type	
Pnma (no. 62)	*a*=5.600, *b*=7.765, *c*=5.394
	V=234.580
Bi 4*c* (0.95)	0.5598 1/4 0.5206
Ag 4*c* (0.05)	0.5598 1/4 0.5206
Fe 4*b* (1.0)	1/2 0 0
O1 4*c* (1.0)	-0.0181 1/4 0.4013
O2 8*d* (1.0)	0.2958 0.0493 0.7007
R-type	*a*=5.427, *c*=13.964
R-3c (no. 167)	V=356.093
Bi 6*a* (0.95)	0 0 ¼
Ag 6*a* (0.05)	0 0 ¼
Fe1 6*b* (1.0)	2/3 1/3 1/3
O 18*e* (1.0)	0.4072 0 1/4
T_1-type	a=5.339, c=4.031
P4/mbm (no. 127)	V=114.889
Bi 2*c* (0.95)	0 1/2 ½
Ag 2*c* (0.05)	0 1/2 1/2

Fe 2*a* (1.0)	0 0 0
O1 2*b* (1.0)	0 0 1/2
O2 4*g* (1.0)	0.1562 0.6564 0
T_2-type	a=5.339, c=8.062
I4/mcm (no. 140)	V=229.773
Bi 4*b* (0.95)	0 1/2 1/4
Ag 4*b* (0.05)	0 1/2 1/4
Fe 4*c* (1.0)	0 0 0
O1 4*a* (1.0)	0 0 1/4
O2 8*h* (1.0)	0.3436 0.8436 0
γ-type	*a*=4.031
Pm-3m (no. 221)	*V*=65.50
Bi 1*b* (0.95)	1/2 1/2 1/2
Ag 1*b* (0.05)	1/2 1/2 1/2
Fe 1*a* (1.0)	0 0 0
O 3*d* (1.0)	0 0 1/2

Table ST1. Atomic spins (in units of Bohr magneton) were obtained by DFT calculations for each of the undoped or Ag-doped phases for FM, AFM-a, AFM-c, and AFM-g spin textures. For undoped crystals Ag atom is not present and so spin on Ag atom is not available (N/A). Data for Alpha and R phases in AFM-c and AFM-g spin configurations are not available as well, as these results could not converge.

	undoped				doped			
	FM	AFM-a	AFM-c	AFM-g	FM	AFM-a	AFM-c	AFM-g
**Alpha**								
Bi	-0.228	0.000	N/A	N/A	-0.126	-0.02	N/A	N/A
Fe	26.536	0.000	N/A	N/A	25.487	0.462	N/A	N/A
O	3.696	0.000	N/A	N/A	2.637	0.379	N/A	N/A
Ag	N/A	N/A	N/A	N/A	0.001	-0.087	N/A	N/A
total/uc	28.004	0.000	N/A	N/A	27.999	0.734	N/A	N/A
**Beta**								
Bi	-0.288	0.000	0.000	0.000	-0.201	0.000	-0.012	0.000

Fe	35.256	0.000	0.000	0.000	34.322	0.000	-0.118	-0.001
O	5.04	0.000	0.000	0.000	3.878	0.000	-0.222	0.000
Ag	N/A	N/A	N/A	N/A	-0.002	0.000	-0.047	0.000
total/uc	40.008	0.000	0.000	0.000	37.997	0.000	-0.399	-0.001
**Gamma**								
Bi	-0.376	0.000	0.000	0.000	-0.203	-0.002	-0.001	0.000
Fe	35.320	0.000	0.000	0.000	34.740	0.004	0.004	0.002
O	4.392	0.000	0.000	0.000	3.494	0.004	0.000	0.000
Ag	N/A	N/A	N/A	N/A	-0.039	0.000	0.000	0.000
total/uc	39.336	0.000	0.000	0.000	37.992	0.006	0.003	0.002
**R**								
Bi	-0.228	0.001	N/A	N/A	-0.125	0.030	N/A	N/A
Fe	26.536	0.000	N/A	N/A	25.488	-0.456	N/A	N/A
O	3.696	0.000	N/A	N/A	2.633	-0.342	N/A	N/A
Ag	N/A	N/A	N/A	N/A	0.001	0.039	N/A	N/A
total/uc	30.004	0.001	N/A	N/A	27.997	-0.729	N/A	N/A
**T1**								
Bi	-0.400	0.000	0.000	0.000	-0.244	-0.063	0.000	-0.011
Fe	35.200	0.000	0.000	0.000	34.211	0.958	-0.001	0.288
O	4.944	0.000	0.000	0.000	4.046	0.880	-0.001	0.132
Ag	N/A	N/A	N/A	N/A	-0.015	0.016	0.000	-0.002
total/uc	39.744	0.000	0.000	0.000	37.998	1.791	-0.002	0.407
**T2**								
Bi	-0.400	0.004	0.000	0.000	-0.244	-0.071	0.000	-0.036
Fe	35.214	0.008	0.000	0.000	34.263	0.975	0.000	0.976

O	4.944	0.000	0.000	0.000	4.001	0.875	0.000	0.712
Ag	N/A	N/A	N/A	N/A	-0.016	0.001	N/A	-0.008
total/uc	39.758	0.012	0.000	0.000	38.004	1.780	0.000	1.644

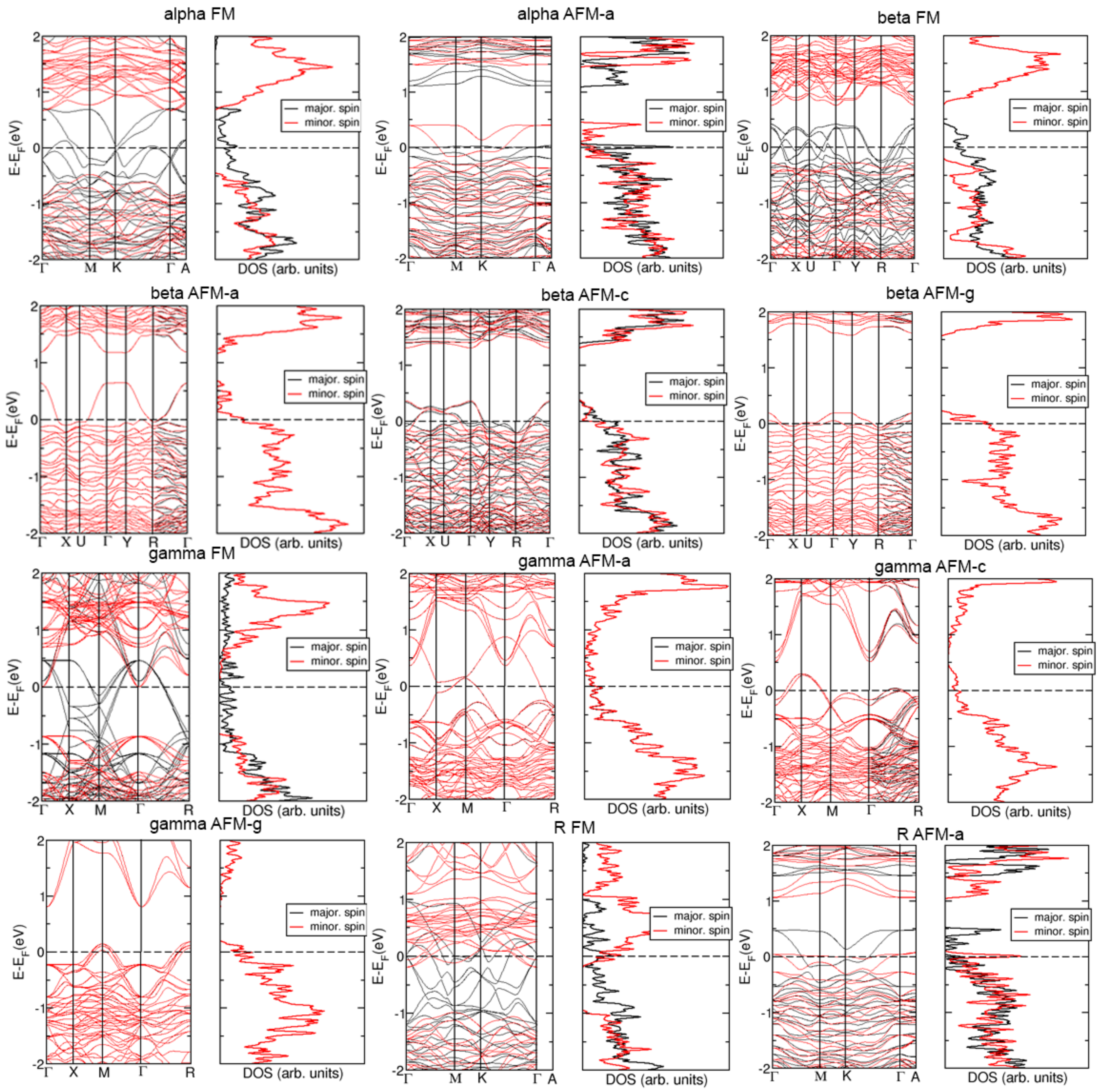

Figure SF1. Electronic band structure and density of states for all combinations of alpha, beta, gamma and R phases on one hand and FM, AFM-a, AFM-c, and AFM-g spin orders on the other hand.

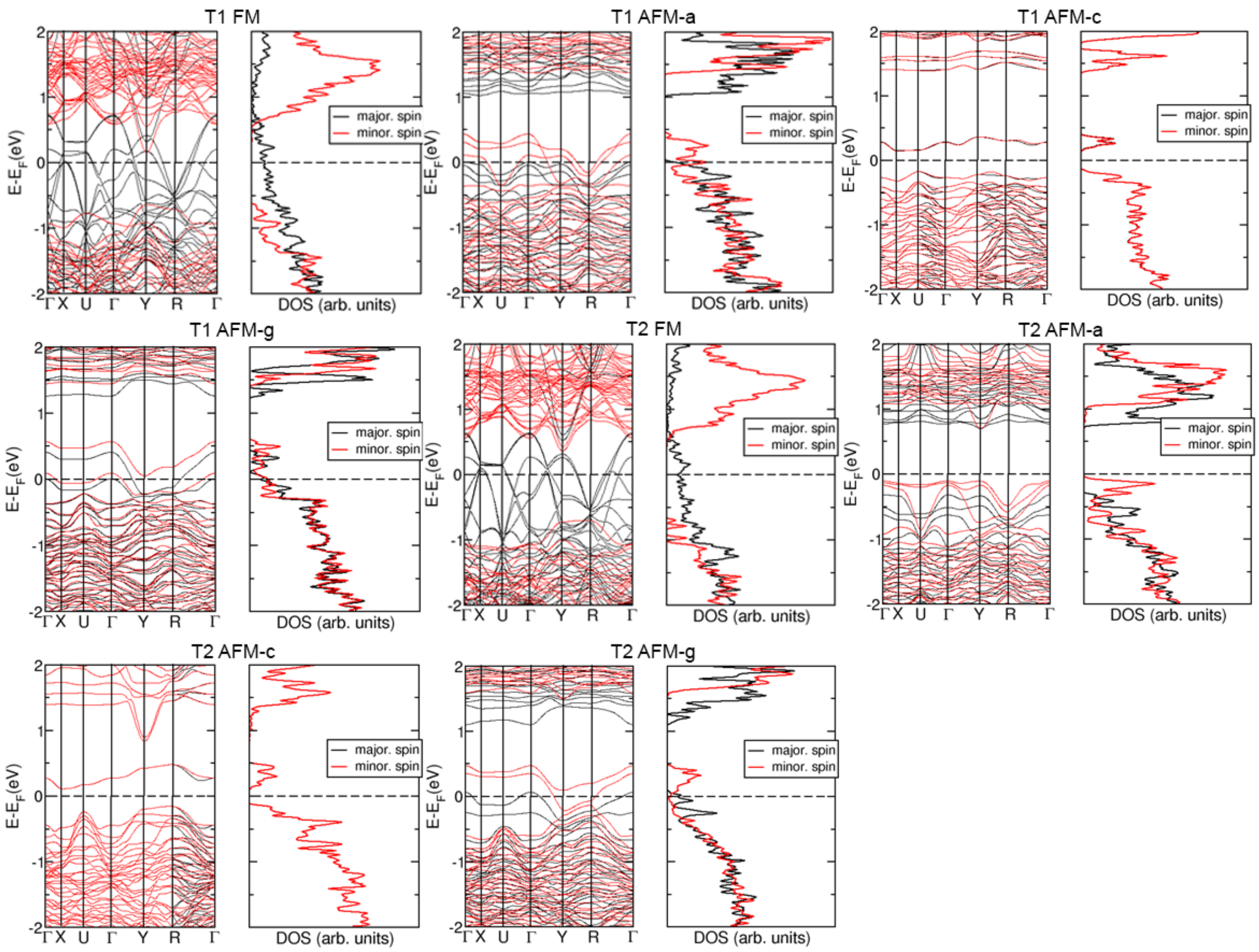

Figure SF2. Electronic band structure and density of states for all combinations of T1 and T2 phases on one hand and FM, AFM-a, AFM-c and AFM-g spin orders on the other hand.
